# Reward learning and statistical learning independently influence attentional priority of salient distractors in visual search

**DOI:** 10.3758/s13414-021-02426-7

**Published:** 2022-01-10

**Authors:** Mike E. Le Pelley, Rhonda Ung, Chisato Mine, Steven B. Most, Poppy Watson, Daniel Pearson, Jan Theeuwes

**Affiliations:** 1 School of Psychology, UNSW Sydney, Sydney, NSW 2052 Australia; 2 Department of Information Technology and Human Factors, Human-Centered Mobility Research Center, National Institute of Advanced Industrial Science and Technology (AIST), Tsukuba, Japan; 3 Japan Society for the Promotion of Science, Tokyo, Japan; 4 Department of Experimental and Applied Psychology, Vrije Universiteit, Amsterdam, The Netherlands

**Keywords:** Attention, Reward, Suppression, Statistical learning, Visual search

## Abstract

Existing research demonstrates different ways in which attentional prioritization of salient nontarget stimuli is shaped by prior experience: Reward learning renders signals of high-value outcomes more likely to capture attention than signals of low-value outcomes, whereas statistical learning can produce attentional suppression of the location in which salient distractor items are likely to appear. The current study combined manipulations of the value and location associated with salient distractors in visual search to investigate whether these different effects of selection history operate independently or interact to determine overall attentional prioritization of salient distractors. In Experiment 1, high-value and low-value distractors most frequently appeared in the same location; in Experiment 2, high-value and low-value distractors typically appeared in distinct locations. In both experiments, effects of distractor value and location were additive, suggesting that attention-promoting effects of value and attention-suppressing effects of statistical location-learning independently modulate overall attentional priority. Our findings are consistent with a view that sees attention as mediated by a common priority map that receives and integrates separate signals relating to physical salience and value, with signal suppression based on statistical learning determined by physical salience, but not incentive salience.

**Supplementary Information:**

The online version contains supplementary material available at 10.3758/s13414-021-02426-7.

Attention refers to the set of cognitive mechanisms that act to prioritize certain aspects of incoming sensory information for further analysis and action and suppress other aspects that might otherwise interfere with our ongoing information processing. A body of research has demonstrated that this prioritization is influenced not only by the sensory properties of stimuli themselves but also by our prior experience and learning (for reviews, see Awh et al., 2012; Failing & Theeuwes, 2018; Le Pelley et al., 2016; Rusz et al., 2020).

The effects of learning on attention can be further subdivided. For example, deployment of spatial attention is influenced by learning about the *location* in which a target is likely to appear, resulting in faster responding to targets appearing there versus another, less-likely location (e.g., Chun & Jiang, 1998; Geng & Behrmann, 2005; Hoffmann & Kunde, 1999; Jiang et al., 2013). Attention is also influenced by learning about the *value* associated with search targets: If responding to a particular stimulus has previously yielded high reward, then attention will prioritize that stimulus in future, relative to a target paired with low reward (e.g., Kiss et al., 2009; Kristjansson et al., 2010; O’Brien & Raymond, 2012; Seitz et al., 2009). The existence of distinct influences of learning on attention—relating to location and value—raises the question of how these influences combine to determine overall priority. This question has been examined in research manipulating both the location and reward received for responding to targets, and the findings of these studies suggest that influences of learning about location and value combine additively to determine activity on a common attentional priority map (Garner et al., 2021; Stankevich & Geng, 2014).

Notably, however, influences of learning on attention are not restricted to the situation in which these properties are associated with targets of search: Learning about the properties of nontarget stimuli also modulates attentional selection. Regarding value, studies have shown that stimuli signalling availability of high reward become more likely to capture attention (relative to stimuli signalling low reward), even if these value-signalling stimuli have never been targets of search (e.g., Kim & Anderson, 2019; Le Pelley et al., 2015; Mine & Saiki, 2015; Pearson et al., 2016; Watson, Pearson, Most, et al., 2019a). The distinction between studies investigating targets versus nontargets is important because effects relating to nontarget stimuli may implicate different attentional systems from those involved in selection of targets. In previous research manipulating value and location of targets (Garner et al., 2021; Stankevich & Geng, 2014), a response to Stimulus X earned a high reward, whereas a response to Stimulus Y earned a low reward. Hence, the reward structure of the task aligned with participants’ goals: Participants could earn more money by prioritizing high-reward stimuli. Under these conditions, prioritization of high-reward targets may reflect a process of top-down, goal-directed selection. By contrast, attentional selection of reward-signalling nontargets is inconsistent with the goal of responding to targets, leading to the suggestion that attentional biases to reward-signalling nontargets reflect the operation of a distinct system of attentional control based on *selection history* (Anderson, 2016; Awh et al., 2012; Failing & Theeuwes, 2018). For example, Watson et al. (2019a; see also Le Pelley et al., 2015) used a variant of the additional singleton task (Theeuwes, 1992), in which participants had to respond to a diamond target among circles on each trial. Correct responses earned reward, and the faster the response, the larger the reward; errors resulted in loss of the amount that would have been won. One of the circles in the search display could be coloured blue or orange; all other shapes were grey. We refer to the colour-singleton circle as the *distractor* to distinguish it from the other (grey) circles in the display. If the distractor appeared in the *high-value* colour (e.g., blue), this signalled that the current trial was a bonus trial on which reward/loss was multiplied by 10; if the distractor was in the *low-value* colour (here, orange), it was not a bonus trial. While the distractor signalled reward magnitude, it was never the target that participants responded to in order to receive that reward. The optimal strategy in this task is to ignore distractors and respond to the target as rapidly as possible, since this would yield highest earnings. However, responses were significantly slower (but not more accurate) for trials with a high-value distractor versus a low-value distractor. This suggests the high-value distractor was more likely to receive attentional priority, interfering with search for the target—even though this behaviour was counterproductive, as it meant participants earned less on high-value trials than would otherwise have been the case.

Such findings demonstrate a qualitatively similar pattern of attentional bias to reward-signalling nontarget stimuli as has previously been shown for targets: In both cases, stimuli associated with high value are prioritized over those signalling low value. By contrast, the effect of learning about stimulus locations is very different for nontargets versus targets. As noted earlier, likely target locations are prioritized by attention—but statistical learning about the properties of distractors results in enhanced attentional *suppression* of salient nontargets (e.g., Stilwell et al., 2019; Wang & Theeuwes, 2018b; for background on the concept of stimulus suppression—though not in the context of learning—see Gaspelin et al., 2015, 2016). Learning about the likely location of a salient distractor stimulus can induce location-specific suppression, such that items appearing in that location are less likely to receive attention than items appearing elsewhere (e.g., Wang & Theeuwes, 2018b, 2018c). For example, Wang and Theeuwes (2018b) used an additional-singleton task in which a colour-singleton distractor appeared most frequently in one location, and less frequently in other locations. Participants were faster to detect the (shape-singleton) target when the search display contained a distractor in the ‘frequent’ location than when the distractor was in one of the ‘rare’ locations. This finding was taken to suggest that statistical learning generated suppression at the frequent-distractor location on the attentional priority map, such that items appearing there competed less for attentional priority than did items at other locations. Consistent with the idea of location-specific suppression, Wang and Theeuwes (2018b, 2018c) also found that on trials without a colour-singleton distractor, responses were slower when the target appeared in the frequent-distractor location than when it appeared in one of the rare locations. Further research has shown that the effect reflects learning about the likely location of distractors rather than the likely location of the target (Failing, Wang, & Theeuwes, 2019b), and is independent of top-down attentional control (Gao & Theeuwes, 2020).

To summarize, learning about the value and location of nontarget (distractor) stimuli modulates attention: regarding value, this modulation is qualitatively similar to that for targets (high-value distractors and targets are prioritized), whereas for location, the effect for targets (prioritization) is opposite to that for distractors (suppression). As noted earlier, influences of value and location have been shown to combine additively to determine attentional priority for targets (Garner et al., 2021; Stankevich & Geng, 2014); given the somewhat different way in which these factors modulate attention with regard to distractors—and the possibility that use of distractors may implicate a distinct system of attentional control based on selection history—a natural question to ask is whether value and location also operate independently in this case. If effects of value and location do indeed represent independent inputs to a combined priority map, then we would expect additive effects regardless of whether the contributing influences are excitatory (prioritization) or inhibitory (suppression). An alternative possibility is that learned suppression is a reactive process, such that high-value (and hence high salience) distractors elicit greater suppression than low-value distractors, which would manifest as an interaction between effects of value and location on overall priority (cf. Failing & Theeuwes, 2020).

Research relevant to this question was recently reported by Kim and Anderson (2021). Their study used a two-phase design. In an initial training phase, participants were rewarded for responding to targets in a reward-related colour and received no reward for targets in a no-reward colour. On half of training trials, the display contained a distractor in a colour not used for the reward and no-reward targets, and this distractor appeared more often in one location than in others. In a subsequent (unrewarded) test phase, participants searched for a shape-singleton target, and a colour-singleton distractor could appear in either the reward or no-reward colour, and in either the frequent-distractor location (from the training phase) or elsewhere. Kim and Anderson found an effect of value on test-phase performance—responses to the target were slower when the display contained a distractor in the reward colour versus the no-reward colour—and an effect of location, with less interference when the distractor appeared in the frequent location than elsewhere. Importantly there was no significant interaction, consistent with additive effects of reward learning and location learning on the processing of distractors under conditions (in the test phase) where attending to distractors was counterproductive, as participants were aware that the target was defined by shape, not colour. However, there are some notable caveats here. First, in Kim and Anderson’s study, value learning and location learning were conducted independently: During training, value information was associated with *targets*, whereas location learning related to a distractor. It is perhaps unsurprising that additive effects should result from such independent training. Second, an analysis of distractor-absent trials in the test phase found no significant difference in performance between trials with a target in the frequent-distractor location versus elsewhere. It is important to demonstrate an effect of location on distractor-absent performance because this provides evidence of location-specific *suppression*. In the absence of this effect, Kim and Anderson’s findings remain open to alternative accounts; for example, a coloured stimulus may be less surprising (and hence interfere less with search) when it appears at the frequent location than at other locations.

## Experiment 1

Experiment 1 investigated further whether prioritization of high-value distractors combines additively with suppression of locations in which those distractors are likely to appear. In contrast to Kim and Anderson (2021), we used a one-phase design in which both value and location information were associated with distractors throughout, thus providing a stronger test of the independence of these influences. Moreover, this one-phase procedure is more like designs that have previously been shown to generate location-specific suppression, as verified by assessing performance on distractor-absent trials (e.g., Wang & Theeuwes, 2018b, 2018c).

Experiment 1 used an additional-singleton task in which participants responded to a shape-singleton target. The colour of a singleton distractor signalled whether a high or low reward was available. Regardless of its colour, this distractor was more likely to appear in one location than in others. We anticipated that the high-value distractor would become more likely to capture attention (and hence slow responding to the target) than the low-value distractor, and that the likelihood of this capture would be reduced when the distractor appeared in its frequent location versus a rare location (suggesting location suppression). The key question was whether these two effects would influence responding independently or would interact.

### Method

#### Participants and apparatus

Previous studies have found medium to large effect sizes (*d*_*z*_ = 0.54–2.20) for the influence of reward on attention in tasks like that used here (e.g., Le Pelley et al., 2015; Watson et al., 2020; Watson, Pearson, Most, et al., 2019a), and large effect sizes (*d*_*z*_ = 0.69–2.15) for the influence of statistical learning about distractor location (Failing, Feldmann-Wüstefeld, et al., 2019a; Failing & Theeuwes, 2020; Failing, Wang, & Theeuwes, 2019b). Consequently, we aimed to recruit at least 29 participants; G*Power (with default settings) revealed that this would give power of .80 to detect medium within-subjects effects (*d*_*z*_ = 0.54). A total of 32 UNSW Sydney students (20 females, 11 males, one other; age *M* = 19.6 years, *SEM* = 0.3) completed the task for course credit, with the top-scoring half of participants also receiving an AU$20 supermarket voucher. Participants completed the experiment online using their web browser; stimulus presentation was controlled by jsPsych (de Leeuw, 2015). All research reported here was approved by the UNSW Sydney Human Research Ethics Advisory Panel (Psychology); experiment code and raw data are available via the Open Science Framework (https://osf.io/zg3nr/).

#### Stimuli and design

**Fig. 1:**
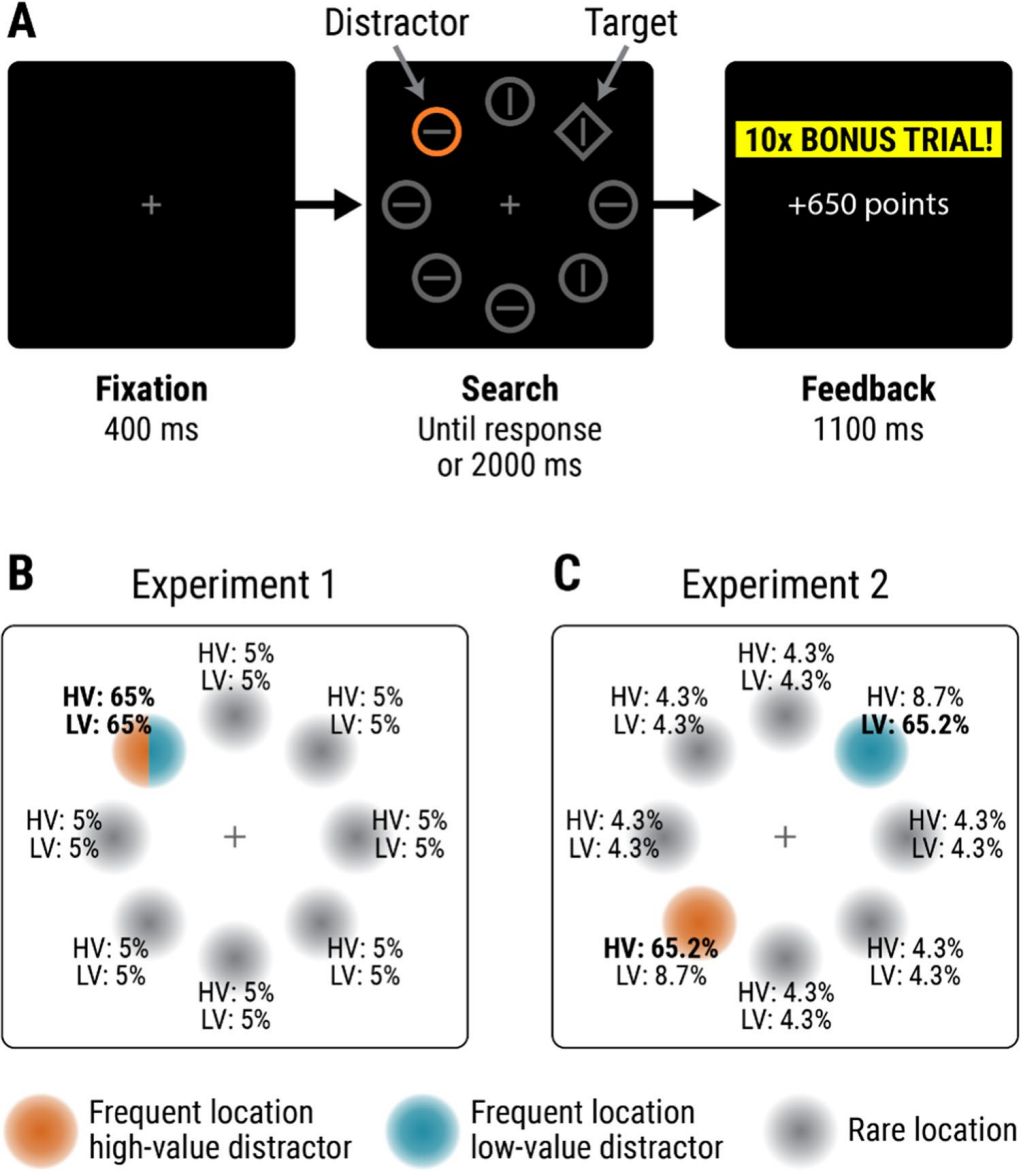
**a** Trial schematic. Participants responded to the orientation of the line in the shape-singleton target: either a diamond among circles (as shown here) or a circle among diamonds. The display could contain a colour-singleton *distractor*, coloured orange or blue. Lower panels illustrate the manipulation of distractor location in (**b**) Experiment 1 and (**c**) Experiment 2. Frequent locations of the high-value (HV) and low-value (LV) distractors are shown in orange and blue, respectively; rare locations are in grey. Percentages at each location give the probability that each distractor type would appear at that location (when that distractor type was present in the display). In Experiment 1, high-value and low-value distractors appeared most frequently at the same location; in Experiment 2, high-value and low-value distractors appeared most frequently at opposite locations. Frequent locations shown here are an example: these locations were chosen randomly for each participant. (Colour figure online)

Each trial (see Fig. 1) began with a central fixation cross on a black background. After 400 ms, the search display appeared. This contained eight shapes (72 × 72 pixels): either one diamond and seven circles or one circle and seven diamonds (randomly determined on each trial), arranged evenly around screen centre at an eccentricity of 140 pixels. Each shape contained a grey (RGB: [70, 70, 70]) line segment oriented horizontally or vertically (randomly). On most trials, one of the non-singleton shapes was coloured either blue (RGB: [37, 141, 165]) or orange (RGB: [193, 95, 30]); all other shapes were grey (RGB: [70, 70, 70]). We term the coloured shape the *distractor*. Assignment of blue and orange to the role of high-value and low-value colours was randomly determined for each participant. Colours were chosen with the intention that the colour-singleton distractor would have higher luminance than the other (grey) display items, thus enhancing the distractor’s physical salience as in our previous work (Pearson et al., 2016; Watson et al., 2020; Watson, Pearson, Most, et al., 2019a), though given the online delivery of this study we cannot be certain how stimuli appeared on participants’ screens.

Participants’ task was to report the orientation of the line in the shape-singleton target as quickly as possible—by pressing either ‘C’ (horizontal) or ‘M’ (vertical)—with faster correct responses earning more points. For trials with a distractor in the low-value colour, or with no colour-singleton distractor (*distractor-absent* trials), correct responses earned 0.1 points per ms that response time (RT) was below 1,400 ms (so an RT of 600 ms earned 80 points). Trials with a high-value distractor were ‘bonus trials,’ with points multiplied by 10 (so an RT of 600 ms earned 800 points). Correct responses with RT above 1400 ms earned no points, and errors resulted in loss of the points that would have been won. RTs below 150 ms were treated as anticipations. The search display remained until a response was made or the trial timed-out (after 2,000 ms). A feedback screen then appeared. If an anticipation had been made, feedback stated, “Please do not anticipate which response to make” for 2,500 ms. In all other cases, feedback appeared for 1,100 ms. If the trial had timed-out, feedback stated “Too slow. 0 points.” Otherwise, if the response was correct, feedback showed the number of points won (e.g., “+80 points”); if the response was incorrect, feedback showed “ERROR” and the number of points lost (e.g., “ERROR: LOSE 350 points”). On trials with a high-value distractor, feedback was accompanied by a box labelled “10× bonus trial!.” Following feedback, the next trial began after a blank intertrial interval of 1,000 ms.

Colour-singleton distractors—regardless of their colour—were more likely to appear in one stimulus location (termed the *frequent* location) than others (*rare* locations). The frequent location was chosen randomly for each participant.

Each block of the task contained 56 trials: 20 trials with a high-value distractor, 20 with a low-value distractor, and 16 distractor-absent trials. Of the 20 trials in each block with the high-value distractor, the distractor appeared 13 times in the frequent location, and once in each of the other seven (rare) locations. The same applied for trials with the low-value distractor. Hence of the trials featuring a distractor, 65% had this distractor in the frequent location and 35% had the distractor in one of the rare locations. Trial order within each block was random, as was the location of the target on each trial.

#### Procedure

Participants were told they should try to earn as many points as possible, with the top-scoring half of participants winning an AU$20 supermarket voucher. As additional motivation, for every 24,000 points earned, participants unlocked a new ‘medal’ (in the order bronze, silver, gold, platinum, diamond, and elite). Based on mean RTs from pilot work, this meant that the best-performing ~10% of participants would unlock the ‘elite’ medal.

Initial instructions stated (1) that faster (correct) responses would earn more points, (2) that when a shape in the high-reward colour appeared in the search display it would be a bonus trial, and (3) that when a shape in the low-reward colour appeared it would not be a bonus trial. Check-questions verified understanding of these instructions: participants had to respond correctly before they could continue. There was no mention that distractors would appear more commonly in one of the locations. Participants then completed 16 blocks (896 trials), taking a break after each block, during which they were shown their total accumulated points, and an animation presented any medals unlocked since the previous break.

Previous studies of statistical learning have examined whether, following the search task, participants were explicitly aware that the distractor appeared more frequently in one location; data relating to (a lack of) awareness have been used to argue that learning of regularities is implicit (e.g., Ferrante et al., 2018; Wang & Theeuwes, 2018b). We remain wary of drawing such conclusions (see General Discussion), but for consistency with previous research we probed participants’ explicit knowledge following the search task. First, participants were asked whether they thought the coloured shape had been equally likely to appear in each of the eight stimulus locations, or if it had been more likely to appear in some location(s) than in others, and rated their confidence in this choice from 1 (*least confident*) to 5 (*most confident*). They were then informed that the coloured shape had been more likely to appear in one of the eight locations than the others, and were asked to select (1) whether this frequent location had been in one of the three upper locations in the search display, one of the two middle locations, or one of the three bottom locations; (2) whether it had been in one of the three left-hand locations, the two central locations, or the three right-hand locations; and (3) in which specific location the distractor had been most likely to appear.

### Results

For two participants, more than a third of search trials had invalid responses (anticipations or time-outs); all data from these participants were excluded from further analyses. After removal of invalid responses, two participants had mean accuracy below 60% and were also excluded. For remaining participants (*n* = 28), following our previous protocols (Le Pelley et al., 2015; Watson, Pearson, Most, et al., 2019a) we discarded data from the first two trials after each break, time-outs (2.6% of all trials), and anticipations (0.2% of all trials); after exclusions, mean accuracy was 82.6% (*SEM* = 1.5%). Analysis of RTs used correct responses only.

#### Distractor-present trials

**Fig. 2:**
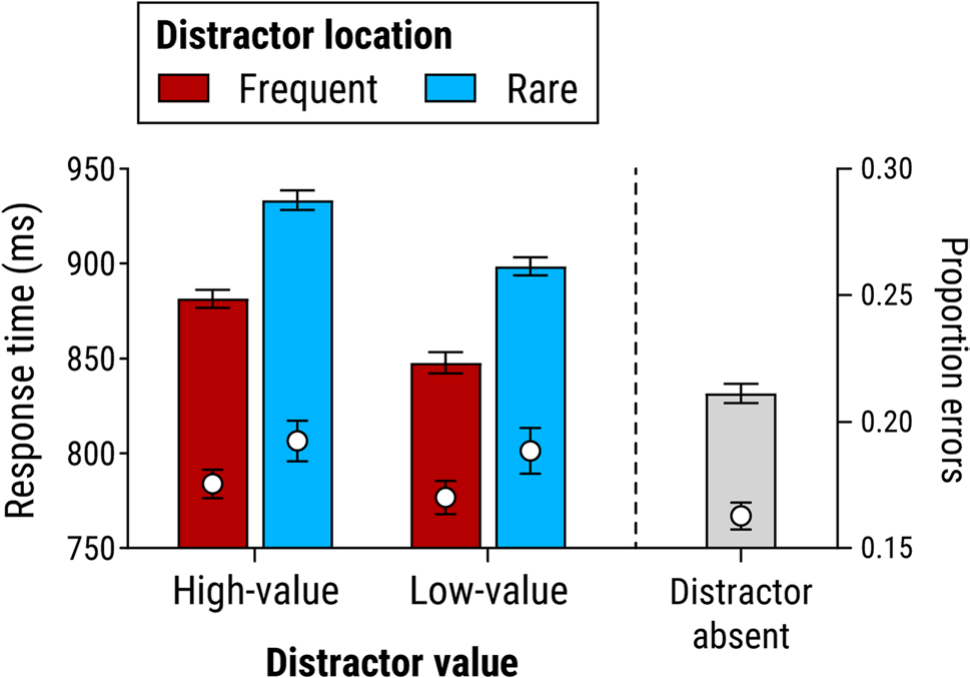
Mean response times and proportion of errors for trials with a colour-singleton distractor in Experiment 1, as a function of the value of the reward signalled by the distractor, and the location of that distractor; and for distractor-absent trials. In this and all other figures, bars show mean response time, superimposed white circles show mean proportion of errors, and error bars show within-subjects standard error of the mean (Morey, 2008)

RT data from trials with a colour-singleton distractor (see Fig. 2) were analyzed via 2 (distractor value: high-value vs. low-value) ×2 (distractor location: frequent vs. rare) analysis of variance (ANOVA). This revealed a main effect of distractor value, *F*(1, 27) = 34.9, *p* < .001, η_*p*_^2^ = .564, with slower responses for high-value than low-value trials. There was also an effect of location, *F*(1, 27) = 88.9, *p* < .001, η_*p*_^2^ = .767, with faster responses when the distractor appeared in the frequent location versus one of the rare locations. Notably, the interaction of value and location was not significant, *F*(1, 27) = 0.04, *p* = .848, η_*p*_^2^ = .001. To assess support for the null hypothesis, we conducted a Bayesian ANOVA using jamovi (The Jamovi Project, 2020) with the default prior. Comparing Bayes factors (*BFs*) for the ‘interactive’ model including both main effects and interaction versus the ‘additive’ model with main effects only, gave *BF* = 3.90 in favour of the additive model. This indicates moderate support for independent effects of value and location (Jeffreys, 1961).

Analysis of errors using similar ANOVA revealed a nonsignificant main effect of value, *F*(1, 27) = 0.31, *p* = .581, η_*p*_^2^ = .011. The main effect of location was significant, *F*(1, 27) = 5.95, *p* = .022, η_*p*_^2^ = .180, with fewer errors when the distractor appeared in the frequent location than a rare location. Again, the Value × Location interaction was nonsignificant, *F*(1, 27) = 0.01, *p* = .907, η_*p*_^2^ < .001, with substantial support for the additive model, *BF* = 4.23.

#### Distractor-absent trials

**Fig. 3:**
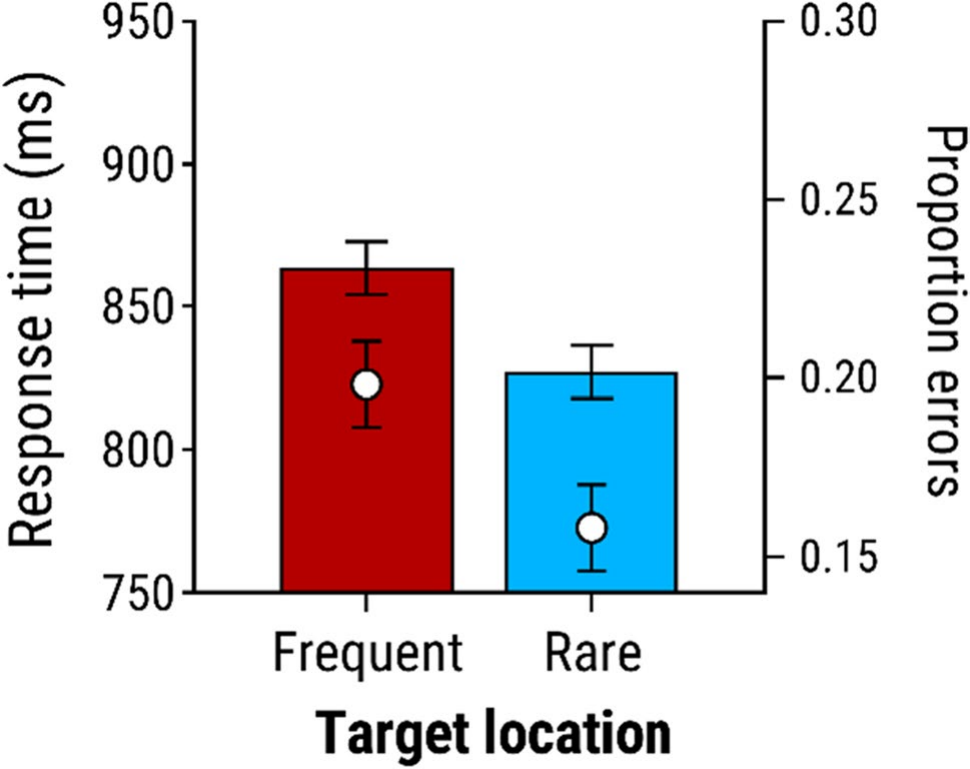
Mean response times and proportion of errors for distractor-absent trials of Experiment 1, as a function of whether the target appeared in the location in which the salient distractor had typically appeared (frequent) versus the average of the locations in which the distractor seldom appeared (rare)

To verify that location-specific suppression had developed at the frequent location, we analyzed performance on distractor-absent trials as a function of the location of the target. Responses were significantly slower, *t*(27) = 2.79, *p* = .010, *d*_*z*_ = 0.527, and less accurate, *t*(27) = 2.35, *p* = .026, *d*_*z*_ = 0.444, when the target appeared in the frequent distractor location than in the average of the rare locations (see Fig. 3).

#### Distractor-present versus distractor-absent trials

Figure 2 (grey bar) shows mean RT and errors collapsed across all distractor-absent trials. Bonferroni-corrected pairwise *t* tests revealed that RT in each of the distractor-present condition was significantly slower than on distractor-absent trials, all *t*(27) ≥ 3.21, *p* ≤ .014, *d*_*z*_ ≥ 0.60. Accuracy was also significantly higher in distractor-absent trials than when the (high- or low-value) distractor appeared in the rare location, both *t*(27) ≥ 2.84, *p* ≤ .034, *d*_*z*_ ≥ 0.54, but not when the distractor was in the frequent location, both *t*(27) ≤ 1.41, *p* ≥ .169, *d*_*z*_ ≤ 0.27. Taken together these findings indicate that the presence of any colour-singleton distractor, regardless of value or location, impaired performance to some degree, indicating that suppression of attentional capture by distractors was not complete in any condition.

#### Awareness of statistical regularities

Of the 28 participants, 18 reported that they thought the distractor had appeared more often in some location(s) than others (versus being random); for these 18 participants, mean confidence was relatively low, at 2.56 (*SEM* = 0.22) on the 5-point scale. After being told that the distractor was more likely to occur in one location, 16 of the 28 participants selected the correct option for the top/middle/bottom position of the frequent location; 20 selected the correct option for the left/middle/right position; and 12 correctly selected the specific frequent location when asked to do so. In each case a binomial test revealed that the proportion of correct choices was significantly greater than chance, *p*s < .05. However, repeating analysis of search RTs while excluding participants who made the correct response regarding the frequent location for each of the knowledge questions left the pattern of significant and nonsignificant results unchanged (see Supplementary Materials for details).

### Discussion

Consistent with previous findings (e.g., Le Pelley et al., 2015; Watson, Pearson, Most, et al., 2019a), responding to the target in Experiment 1 was slower when the search display contained a high-value distractor versus a low-value distractor. This implies that participants were more likely to attend to the high-value distractor, interfering with search for the target. This effect of distractor value was counterproductive, since response times influenced the points earned in the search task: By responding more slowly (but no more accurately) on trials in which higher rewards were available, participants lost out disproportionately.

Moreover, search performance was significantly better (faster and more accurate) when the colour-singleton distractor appeared in the frequent location than in a rare location. This finding is again consistent with prior research (e.g., Wang & Theeuwes, 2018b, 2018c) and has been taken to reflect the development of attentional suppression at the frequent location, such that salient items presented at this location compete less for attentional priority than items at other locations. In line with this interpretation, we also found that on distractor-absent trials, participants were slower to respond to the target when it appeared in the frequent-distractor location versus a rare-distractor location. This latter finding confirms that location-specific suppression had developed at the frequent location.

Importantly, the effects on performance of distractor value and location were additive: there was no significant interaction between these factors, and Bayesian analyses supported the null hypothesis. This pattern is consistent with the findings of Kim and Anderson (2021) in suggesting the independence of value-learning and location-learning effects on the processing of distractors under conditions in which attending to distractors was counterproductive, as participants were aware that the target was defined by shape, not colour. Notably (and unlike Kim & Anderson), Experiment 1 demonstrates this pattern in a procedure in which both value and location were associated with distractor stimuli throughout the task. Additive effects under these conditions hence constitute a strong demonstration of independence. Moreover, we found an effect of target location on performance on distractor-absent trials, providing stronger evidence of spatial *suppression* resulting from statistical learning, and hence its independence from effects of value—bolstered by results of Bayesian analysis.

The implication is that reward learning and statistical learning exert independent effects on attentional prioritization of distractors, consistent with these processes having separate inputs to a common attentional priority map. High-value distractors were more salient than were low-value distractors (they interfered more with search for the target) but did not elicit greater suppression. This finding is in line with the idea of suppression being proactive: On this account, a fixed, ‘negative priority’ input acts at the frequent location prior to appearance of the search display and subtracts from the salience of whatever item appears at that location. By contrast, our findings are harder to reconcile with the idea that suppression is driven by reaction to the salience of a presented distractor—or at least suggest that any such reactive suppression is subject to a low ceiling, such that presentation of a high-value distractor swamps the limited suppression that can be applied.

## Experiment 2

Experiment 1 examined the case in which there was a single location in which both types of distractors—high-value and low-value—were most likely to appear. Participants did not know which type of distractor would appear on a given trial, and under these conditions it makes sense that the attentional system might develop a suppression of the frequent location that would apply equally regardless of the value (and colour) of the distractor presented at that location (a similar argument applies to Kim & Anderson, 2021). In Experiment 2, we instead used a search task in which high-value distractors were likely to appear in one location (termed the *frequentHigh* location), while low-value distractors were likely to appear in a second location (*frequentLow*), to investigate whether this difference in experience would lead to different levels of suppression at the frequentHigh and frequentLow locations. This procedure provides a particularly strong test of the putative independence of reward-related attentional prioritization and spatial suppression of frequent distractor locations, because it represents a design in which the two manipulations are tightly integrated. That is, under these conditions there should be a greater drive to develop suppression at the frequentHigh location, since this location will typically hold very salient high-value distractors, and suppressing attention to these distractors is particularly beneficial because it would result in larger rewards under the task’s payoff structure. By contrast, if effects of reward learning and statistical learning on attention are independent (as suggested by the results of Experiment 1), then we should expect to see equal evidence of suppression at both frequentHigh and frequentLow locations.

### Method

Method was as for Experiment 1, with exceptions as noted here. A total of 44 participants (14 females, 28 males, two other; age *M* = 27.1 years, *SEM* = 1.2) were recruited via Prolific (www.prolific.co) and received £7.50 for participating, with the top-scoring 25% of participants receiving a bonus of £4. For each participant, one of the eight stimulus locations was randomly chosen to serve as the frequentHigh location; the frequentLow location was in the diametrically opposite position.

Each block contained 56 trials: 23 trials with the high-value distractor, 23 with the low-value distractor, and 10 distractor-absent trials. Of the 23 trials in each block with the high-value distractor, it appeared 15 times in the frequentHigh location, twice in the frequentLow location, and once in each of the other six (rare) locations. Likewise, of the 23 trials with the low-value distractor, it appeared 15 times in the frequentLow location, twice in the frequentHigh location, and once in each of the other six (rare) locations. Consequently, each distractor appeared 65.2% of the time in its own most frequent location, 8.7% of the time in the most frequent location of the other distractor, and 4.3% of the time in each of the other six locations. Target location was random on distractor-present trials but was constrained for distractor-absent trials; of the 10 distractor-absent trials in each block, the target appeared twice in each of the frequentHigh and frequentLow locations, and once in each of the other six locations. Participants completed 16 blocks (896 trials total).

Following the search task, as in Experiment 1, participants were asked whether they thought the coloured shape had been equally likely to appear in each of the eight stimulus locations, or if it had been more likely to appear in some location(s) than others and rated their confidence in this choice. Participants were then informed that the blue shape had been most likely to appear in one location, and the orange shape in a different location. They were then asked to indicate in which location each type of distractor (high-value and low-value colours) had been most likely to appear, in random order.

### Results

No participants had more than a third of trials in the search task with invalid responses. After removal of invalid responses, three participants had mean accuracy below 60% and were excluded from subsequent analyses. For remaining participants (*n* = 41), we discarded data from the first two trials after each break, time-outs (0.79% of all trials), and anticipations (0.04% of all trials); after exclusions, mean accuracy was 86.4% (*SEM* = 0.8%).

#### Distractor-present trials

**Fig. 4:**
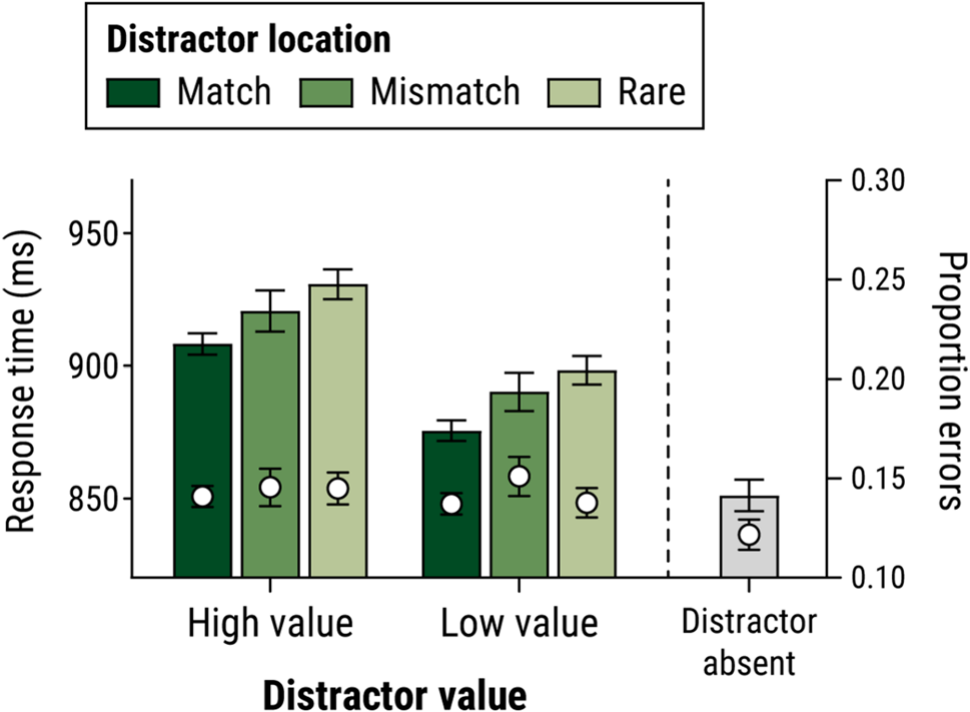
Mean response times and proportion of errors for trials with a colour-singleton distractor in Experiment 2, as a function of the reward value of the distractor, and its location: whether the distractor appeared in its own most frequent location (i.e., high-value distractor in frequentHigh location; low-value distractor in frequentLow location: labelled *match*), or in the most frequent location of the other type of distractor (high-value distractor in frequentLow location and vice versa: *mismatch*), or in one of the rare locations. For comparison, the figure also shows mean performance on distractor-absent trials

Figure 4 shows mean RT (for correct responses) and error rate for trials with a colour-singleton distractor. Data are grouped according to whether the distractor appeared in its own most frequent location (i.e., high-value distractor in frequentHigh location; low-value distractor in frequentLow location: labelled the *match* condition), or in the most frequent location of the other type of distractor (high-value distractor in frequentLow location and vice versa: *mismatch* condition), or in one of the rare locations. For this analysis, we excluded trials in which the target appeared at either the frequentHigh or frequentLow location (i.e., for all trials in this analysis, the target appeared at a ‘rare’ location).

RT data were analyzed via 2 × 3 ANOVA, with factors of distractor value (high-value vs. low-value) and location (match, mismatch, rare). This revealed a main effect of distractor value, *F*(1 ,40) = 27.0, *p* < .001, η_*p*_^2^ = .403, with slower responses for high-value than for low-value trials, and a main effect of location, *F*(2, 80) = 8.88, *p* < .001, η_*p*_^2^ = .182. Tukey HSD tests revealed that responses were significantly faster in the match condition than the mismatch condition, *t*(80) = 2.50, *p* = .038, and in the match condition than in the rare condition, *t*(80) = 4.19, *p* < .001; there was no significant RT difference between mismatch and rare conditions, *t*(80) = 1.69, *p* = .217. Returning to the ANOVA, there was no significant Value × Location interaction, *F*(2, 80) = 0.03, *p* = .973, η_*p*_^2^ < .001; that is, the pattern of suppression across match, mismatch, and rare locations did not differ significantly depending on whether a high-value or low-value distractor appeared. Bayesian ANOVA revealed *BF* = 13.1 in favour of an additive model (main effects only) over an interactive model, indicating strong evidence for independent effects of value and location (Jeffreys, 1961). ANOVA analysis of error rates revealed no significant main effects or interaction, all *F*s < 1.

#### Distractor-absent trials

**Fig. 5:**
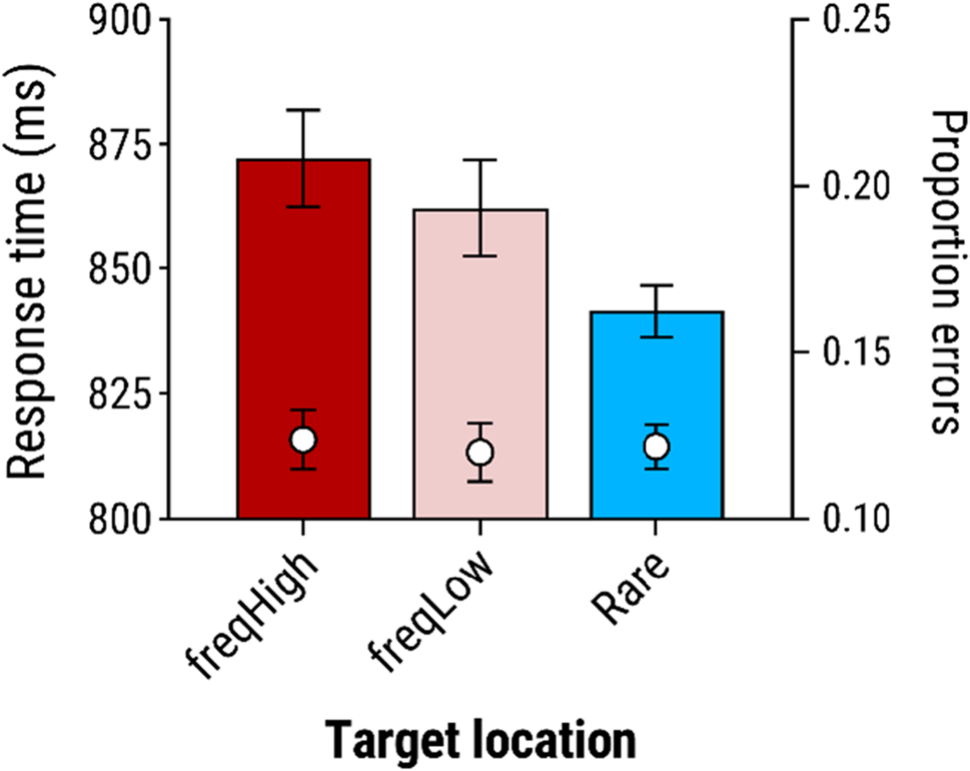
Mean response times and proportion of errors for distractor-absent trials of Experiment 2, as a function of whether the target appeared in the location in which the high-value distractor had typically appeared (freqHigh), or the location in which the low-value distractor had typically appeared (freqLow), versus the average of the remaining six locations in which the distractors seldom appeared (rare)

To verify that location-specific suppression had developed at the frequent locations, we used one-way ANOVA to analyze RT on distractor-absent trials as a function of whether the target appeared at the frequentHigh location, the frequentLow location, or one of the rare locations (see Fig. 5). This revealed a significant effect of target location, *F*(2, 80) = 3.44, *p* = .037, η_*p*_^2^ = .079. Planned *t* tests revealed that responses were significantly faster when the target was in a rare location versus the frequentHigh location, *t*(40) = 3.12, *p* = .003, *d*_*z*_ = 0.487, or the frequentLow location, *t*(40) = 2.07, *p* = .045, *d*_*z*_ = 0.322. There was no significant RT difference when the target was in the frequentHigh versus frequentLow location, *t*(40) = 0.66, *p* = .513, *d*_*z*_ = 0.103, with a Bayesian *t* test indicating substantial support for the null, *BF*_01_ = 4.83. ANOVA analysis of corresponding error data revealed no significant effect of target location, *F*(2, 80) = 0.06, *p* = .946, η_*p*_^2^ = .001.

#### Distractor-present versus distractor-absent trials

Figure 4 (grey bar) shows mean RT and errors across all distractor-absent trials. Bonferroni-corrected *t* tests revealed significantly slower RT in each of the distractor-present conditions than on distractor-absent trials, all *t*(40) ≥ 5.69, *p* ≤ .001, *d*_*z*_ ≥ 0.88. Thus, the presence of any colour-singleton distractor, regardless of value or location, impaired performance to some degree, indicating that suppression of attentional capture by distractors was not complete in any condition. Accuracy did not differ significantly on distractor-absent trials versus any of the distractor-present conditions, all *t*(40) ≤ 2.52, *p* ≥ .096, *d*_*z*_ ≤ 0.39.

#### Awareness of statistical regularities

Of the 41 participants, 16 reported that they thought the distractors had appeared more often in some location(s) than others (versus being random); for these 16 participants, mean confidence was moderate, at 3.38 (*SEM* = 0.18) on the 5-point scale. After being told that each distractor colour was most likely to occur in a distinct location, 21 of the 41 participants correctly identified the frequentHigh location for the high-value distractor, and 18 correctly identified the frequentLow location for the low-value distractor. Binomial tests revealed that performance for both types of distractors was significantly above chance, *p*s < .001, and a McNemar test indicated that selection of the correct location for the high-value versus low-value distractor did not differ significantly, McNemar χ^2^(1) = 0.69, *p* = .405. Repeating analysis of search RTs while excluding the 13 participants who correctly chose both frequent locations did not change the overall pattern of significant main effects of distractor value and location, with no significant interaction (see Supplementary Materials).

### Discussion

In Experiment 2, high-value and low-value distractors typically appeared in distinct locations. Under these conditions, we again found that search performance was impaired when the display contained a high-value distractor versus a low-value distractor—indicating an influence of reward learning on attentional priority—and evidence of suppression resulting from statistical learning about frequent distractor locations, with faster responses when the distractor appeared in its most frequent location (frequentHigh for the high-value distractor; frequentLow for the low-value distractor) versus one of the rare locations. Critically, however, we again found no evidence of an interaction between effects of value and location, with Bayesian analysis providing strong support for additive effects. This is notable because the design of Experiment 2 should have created a greater drive to suppress items appearing at the frequentHigh location, since suppressing high-reward distractors—which were most common at this location—was most beneficial for earning reward in this task. Nevertheless, no difference in suppression was observed at the frequentHigh and frequentLow locations.

Consistent with prior research (Failing, Feldmann-Wüstefeld, et al., 2019a; see also Stilwell et al., 2019; Vatterott & Vecera, 2012) there was some evidence that the (reward-independent) suppression observed in Experiment 2 was feature specific, in that responses were faster when the colour of the distractor on a given trial matched the colour of the distractor that most frequently appeared at that location, than when it mismatched (e.g., search performance was better when a blue distractor appeared at a location in which a blue distractor had appeared most frequently, versus when it appeared at a location in which an orange distractor had appeared most frequently). That said, however, the suppression was not *entirely* feature specific. As in Experiment 1, responses on distractor-absent trials were slower when the target appeared in one of the frequent distractor locations versus one of the rare locations. This latter finding suggests that, in addition to a feature-specific suppression of a particular colour at a particular frequent-distractor location, there was also a feature-general suppression that applied to any item presented at that location. One slightly discrepant result in this regard is that we did not see a significant difference in performance on distractor-present trials in the mismatch versus rare conditions: If there were some degree of general suppression at both frequent-distractor locations, then we should expect to see faster responding in the mismatch condition. The null result here may simply reflect noise in the data (mismatch trials were the least frequent and hence had greatest noise); we note that Fig. 4 shows a (nonsignificant) numerical trend towards a mismatch-vs.-rare difference, and Failing, Feldmann-Wüstefeld, et al. (2019a) did find a significant mismatch-vs.-rare difference in their study which was considerably longer than ours (2,304 trials versus 896 in the current study, providing greater signal-to-noise ratio) but which did not manipulate reward.

Failing, Feldmann-Wüstefeld, et al. (2019a) took their data indicating both feature-specific and feature-general suppression—which mirror the current findings—as evidence that learned suppression operates on multiple levels of the visual processing hierarchy: At the level of *feature maps* (e.g., a saliency map for the colour blue and a separate map for orange), and *conspicuity maps* (e.g., a colour map, which sums the individual feature maps for specific colours). Information from conspicuity maps for different dimensions (colour, shape, etc.) is then fed forward to an overall priority map (Itti & Koch, 2000, 2001). On this account, when a blue singleton appears in a location in which a blue distractor has often appeared in the past (match condition), it is subject to suppression both on the blue feature map and the colour conspicuity map. By contrast, when a blue singleton appears in a location in which an orange distractor has appeared in the past (mismatch condition), it receives suppression only at the level of the conspicuity map. Hence this approach correctly anticipates greater suppression in the match condition than the mismatch condition (for further detail, see Failing, Feldmann-Wüstefeld, et al., 2019a; see also Gaspelin & Luck, 2018a, for related research).

## General discussion

Experiments 1 and 2 found clear evidence of effects of both distractor value and location on participants’ ability to respond to a search target. Critically, these effects were additive, suggesting that the two factors exert independent effects on attentional priority. In Experiment 1, high-value and low-value distractors were most likely to occur in a single location. In this case, the independent effects of value and location indicate that a fixed amount of suppression was applied to this frequent location, and this suppression applied equally to both types of distractors (consistent with recent findings reported by Kim & Anderson, 2021). Consequently, high-value distractors were more likely to ‘break through’ this suppression and capture attention, thus slowing search—an idea that is consistent with conclusions from previous research examining effects of reward in the context of other forms of inhibition (Pearson et al., 2020; Wang et al., 2014). In Experiment 2, high-value and low-value distractors were most likely to occur in different locations, creating a situation in which there should have been a greater drive to develop suppression at the frequent location of high-value distractors (since this would allow greater earnings on the most important, high-value trials). Nevertheless, once again, the degree of suppression was uninfluenced by reward value: Results indicated that a similar level of suppression applied at the frequent locations of high-value and low-value distractors.

Previous research has found additive effects of knowledge regarding value and location associated with targets of search: Participants prioritize locations that are likely to hold targets, and features of stimuli that yield high reward when responded to as targets (Garner et al., 2021; Stankevich & Geng, 2014). Our findings show that this independence of value and location effects extends to the case in which these properties are associated with distractor stimuli that have never been explicit targets of search. This is notable because prioritization of high-value distractors under these conditions is counterproductive: Attending to distractors slowed responses to the target and hence reduced earnings, and participants knew that the best strategy was to ignore the coloured shapes altogether and simply respond as quickly as possible on every trial. Such findings have been taken to suggest that the value-related attentional bias is not a product of strategic, goal-directed control, but instead reflects an influence of *selection history* on the likelihood that stimuli will capture attention (Awh et al., 2012; Belopolsky, 2015; Failing & Theeuwes, 2018). That said, we should consider the alternative possibility that participants monitored for the high-value distractor strategically because it had *informational value* (Gottlieb et al., 2014)—it signalled when a large reward was available—even though this was a poor strategy to use since it would result in lower earnings. This latter interpretation is unlikely, however. First, previous work has shown that the attentional bias to the high-value distractor persists even when all rewards are removed, such that distractors no longer have any informational value and hence there is no strategic reason to select them (Watson, Pearson, Most, et al., 2019a; see also Mine & Saiki, 2015). Second, the current data show that suppression was applied at the location of both the high-value and low-value distractors, indicating a drive to *prevent* attention to these distractors, rather than to attend to them and make use of the value information that they provided. Hence, we believe the effect of value on performance in the current task reflects an influence of selection history on attention, wherein signals of high-value outcomes come to be automatically prioritized, rather than strategic selection. Consistent with previous theorizing in this area (e.g., Anderson et al., 2011; Failing & Theeuwes, 2014; Mine & Saiki, 2015), in this article we have interpreted the influence of value in terms of its effect on attentional *capture,* wherein high-value distractors are more likely to be selected than low-value distractors. However, previous work has also shown that value can also exert a counterproductive—and apparently nonstrategic—influence on attentional *disengagement* (people are slower to move their attention away from high-value distractors than low-value distractors; Watson et al., 2020; see also Müller et al., 2016), and such an influence may have contributed to the pattern of slower responding on trials with a high-value versus low-value distractor in the current study. While we can conclude that there was attentional prioritization of high-value distractors, the response-time measure used here does not allow us to disentangle the degree to which this selection-history bias reflects an effect of value on capture versus disengagement.

Regardless, the finding of additive effects of value and location on attentional prioritization of distractors underlines the independence of these two contributions to the common attentional priority map. Our results demonstrate that this independence extends beyond the case in which the task structure is aligned with participants’ goals (Garner et al., 2021; Stankevich & Geng, 2014)—such that behaviour may be purely mediated by the goal system—and extends to the operation of an alternative system of attentional control based on selection history.

Moreover, by attaching location information to distractors—rather than targets—our study moves from a situation in which statistical location-learning leads to attentional prioritization to one in which the outcome is attentional suppression. Previous work on suppression has led to the development of the *signal suppression hypothesis*, which proposes that salient distractor items can activate an inhibitory attentional mechanism that tends to suppress attentional capture (Gaspelin et al., 2015, 2016; Gaspelin & Luck, 2018a, 2018b). Gaspelin and Luck (2018b) suggested that items are suppressed based on their simple, physical features (e.g., colour; see also Stilwell et al., 2019; Vatterott & Vecera, 2012) and raised as an outstanding question the issue of whether suppression would also be influenced by other factors that might render a stimulus salient. The current findings address this issue by demonstrating that suppression is not influenced by salience that derives from the value of a stimulus ('incentive salience': see Berridge & Robinson, 1998; Colaizzi et al., 2020), rather than its physical features. Thus, our findings are consistent with the idea that suppression develops at the level of feature/conspicuity maps relating to physical features (see also Failing, Feldmann-Wüstefeld, et al., 2019a; Gaspelin & Luck, 2018a), rather than in maps relating to value, or in response to salience in an integrated, common priority map (since in that case we would expect greater suppression at the location of the higher salience, high-value distractors).

In line with this suggestion, the results of Experiment 2 stand in notable contrast to those of a recent study by Failing and Theeuwes (2020), who investigated the effect of *physical* salience on suppression by varying the distinctiveness of a colour-singleton distractor relative to other items in the search display. Some trials of their task featured a high-salience distractor (a red shape that was very distinct from the other, green items in the display), whereas other trials featured a low-salience distractor (a yellowish-green shape). Following the general approach of the current Experiment 2, the high-salience distractor was most likely to appear in one location, and the low-salience distractor was most likely to appear in a different location. Critically—and unlike the current study—Failing and Theeuwes found that this manipulation of physical salience generated stronger suppression in the frequent location of the high-salience distractor than in the frequent location of the low-salience distractor. Hence, it seems that differences in physical salience of distractors can result in different levels of suppression arising from statistical learning, but differences in value do not. Again, this conclusion is consistent with the idea that suppression develops at the level of salience maps relating to physical features, rather than maps encoding value (Gaspelin & Luck, 2018b; Stilwell et al., 2019; Vatterott & Vecera, 2012).

In each of the current experiments, many participants failed to correctly identify the location in which distractors had most frequently appeared when asked to do so. Restricting analyses to only these participants revealed a similar pattern of findings to overall analyses (main effects of value and location, with no interaction). Such findings have previously been taken as evidence of implicit learning of statistical regularities, and by extension that the resulting suppression is not mediated by goal-directed control based on explicit awareness, but instead reflects attentional control driven by selection history (e.g., Ferrante et al., 2018; Wang & Theeuwes, 2018b; see also Wang & Theeuwes, 2018a). While our findings are in line with this view, we are wary of interpreting a participant’s failure to select the correct location in a ‘one-shot’ awareness test at the end of a long experiment—when motivation is likely to be low—as evidence of a total lack of explicit knowledge (see Lovibond & Shanks, 2002; Vadillo et al., 2016). It seems likely that such tests do not provide an exhaustive assessment of relevant conscious knowledge and are not as sensitive in assessing that knowledge as is the test of effects on performance (based hundreds or thousands of search trials). Hence, failure on such awareness tests may not be as diagnostic of implicit processes as previous work has suggested. We remain agnostic on this issue and note that the question of (un)awareness was not central to the current study.

Our study has some limitations. First, the experiments were run online, as a consequence of COVID-19 preventing in-person testing. This reduced our degree of control over stimulus presentation (size, luminance, etc.) and testing conditions (distractions while participants were performing the task), which may have created additional noise in the data. For instance, mean error rates were around 15%–20%, which was somewhat higher than in similar laboratory-run studies (e.g., 7%–8% in Kim & Anderson, 2021; Wang & Theeuwes, 2018a), perhaps a consequence of online testing. Nevertheless, all manipulations were within subjects, and both experiments found clear evidence of effects of distractor value and location on performance—with large effect sizes—demonstrating that our online implementation of the search task was sensitive to detecting effects of the critical independent variables on performance. Second, in these experiments the presence of a high-value distractor (versus a low-value distractor) signalled that a correct response to the target would earn larger reward and also that an error would lead to a larger loss of points. Under these conditions, we cannot be sure whether the observed influence of value on attention reflects an effect of the stimulus’s relationship with reward, with punishment, or both. Based on prior research, it seems likely that both have an effect: that the critical issue is the motivational significance of the outcome signalled by a stimulus rather than the valence of that outcome (see Watson, Pearson, Wiers, & Le Pelley, 2019b). For current purposes it was sufficient to show that performance in this task was influenced by ‘value’—which incorporates both reward and punishment—but future research could study this issue more closely using a procedure in which correct responses are rewarded but errors are not punished (to investigate the effect of reward learning specifically), or in which errors are punished but correct responses are not rewarded (to investigate the effect of punishment).

One further feature of the current study worth considering is that value learning and statistical learning related to different stimulus dimensions: value-related prioritization was based on colour, whereas learned suppression was tied to location (see also Kim & Anderson, 2021). We have interpreted our findings as suggesting additive effects of selection history mechanisms relating to value learning and learned suppression, but we should consider the alternative (and more specific) possibility that the additivity instead reflects the operation of distinct and noninteracting attentional systems relating to featural (colour) and spatial (location) dimensions. This latter account cannot be the whole story, however, because the basic effect of learned suppression from statistical learning (as demonstrated by e.g., Wang & Theeuwes, 2018b, 2018c, and in the current study) shows that suppression of a *location* arises as a result of experience of where a *colour* appears. In other words, the distractor-suppression effect demonstrates that learning about colours *can* influence attention to locations, indicating in turn that featural and spatial systems are not entirely isolated from one another. Consequently, we believe that the additive effects observed in the current study do not simply reflect distinct systems for colour and location, but instead suggest a more general independence between effects of value learning and learned suppression on attentional prioritization. Nevertheless, it remains possible that more evidence for interaction between value learning and learned suppression would be observed if both properties were associated with the same stimulus dimension; this remains an issue for future research.

In summary, the current study investigated two factors known to shape attentional prioritization of salient distractors in visual search—learning about the value of outcomes signalled by these distractors, and the location in which they are likely to appear. Our results indicate that these influences are additive, suggesting that learning about value and learning about location exert independent effects on attention to distractors—a finding which can be reconciled with an account in which attentional priority is mediated by a common priority map that receives and integrates signals relating to physical salience and reward. More generally, our findings sit within a growing body of research indicating that attention is not simply a function of stimulus features and top-down goals but is instead critically influenced by our prior experiences—and extends this idea by showing that different effects of prior experiences may themselves be mediated by distinct cognitive pathways.

## Supplementary Information

ESM 1 (DOCX 154 kb)
