# Supplementary material for: Reward learning and statistical learning independently influence attentional priority of salient distractors in visual search
Source: Atten Percept Psychophys. 2022 Jan 10;84(5):1446–59. doi: 10.3758/s13414-021-02426-7 (PMC8747445; doi:10.3758/s13414-021-02426-7)
Supplement: Supplementary file 1 — (DOCX 154 kb) [file 13414_2021_2426_MOESM1_ESM.docx]

**SUPPLEMENTARY MATERIALS TO ACCOMPANY**

**Reward learning and statistical learning independently influence**

**attentional priority of salient distractors in visual search**

Mike E. Le Pelley^1^, Rhonda Ung^1^, Chisato Mine^1,2,3^, Steven B. Most^1^,

Poppy Watson^1^, Daniel Pearson^1^, & Jan Theeuwes^4^

1. School of Psychology, UNSW Sydney, Australia
2. Human-Centered Mobility Research Center, Department of Information Technology and Human Factors, National Institute of Advanced Industrial Science and Technology (AIST), Tsukuba, Japan
3. Japan Society for the Promotion of Science
4. Department of Experimental and Applied Psychology, Vrije Universiteit, Amsterdam, The Netherlands.

**Corresponding author:**

Prof Mike Le Pelley

School of Psychology, UNSW Sydney

Sydney NSW 2052

Australia

This document presents analyses of search performance in participants who, in the test of explicit awareness at the end of the experiment, did not report the correct location(s) in which the distractor(s) frequently appeared during the search task.

# Experiment 1

In the test of explicit awareness following the search task of Experiment 1, participants were asked to select (1) whether the frequent distractor location had been in one of the three upper locations in the search display, one of the two middle locations, or one of the three bottom locations; (2) whether it had been in one of the three left-hand locations, the two central locations, or the three right-hand locations; and (3) in which specific location the distractor had been most likely to appear. For each of these questions, we report below the results of analyses of the critical search data that were restricted to participants who made an incorrect response regarding the frequent distractor location; that is, participants who did not demonstrate explicit awareness of the frequent location.

## Top/Middle/Bottom Question

Of the 28 participants, 12 chose the incorrect top/middle/bottom location of the frequent distractor. Figure S1A shows response time (RT) and error data for these 12 participants, for trials with a colour-singleton distractor. These data were analysed via 2 (distractor value: high-value vs. low-value) ×2 (distractor location: frequent vs. rare) ANOVA. Analysis of RT data revealed a main effect of distractor value, *F*(1,11) = 13.9, *p* = .003, η*_p_*^2^ = .558, with slower responses for high-value than low-value trials. There was also an effect of location, *F*(1,11) = 22.4, *p* < .001, η*_p_*^2^ = .671, with faster responses when the distractor appeared in the frequent location versus one of the rare locations. The interaction of value and location was not significant, *F*(1,11) = 0.08, *p* = .788, η*_p_*^2^ = .006. Analysis of error data revealed no significant effects, all *F*(1,11) ≤ 1.35, *p* ≥ .269.

## Left/Central/Right Question

Of the 28 participants, 8 chose the incorrect left/central/right location of the frequent distractor; Figure S1B shows data for these participants. ANOVA analysis of RT data revealed significant main effects of distractor value, *F*(1,7) = 5.83, *p* = .046, η*_p_*^2^ = .454, and location, *F*(1,7) = 31.8, *p* < .001, η*_p_*^2^ = .820, but no significant interaction, *F*(1,7) = 0.04, *p* = .838, η*_p_*^2^ = .006. Analysis of error data revealed no significant effects, all *F* < 1.

## Specific Location Question

Of the 28 participants, 16 chose the incorrect specific location of the frequent distractor; Figure S1C shows data for these participants. ANOVA analysis of RT data revealed significant main effects of distractor value, *F*(1,15) = 17.7, *p* < .001, η*_p_*^2^ = .540, and location, *F*(1,15) = 36.3, *p* < .001, η*_p_*^2^ = .708, but no significant interaction, *F*(1,15) = 0.27, *p* = .609, η*_p_*^2^ = .018. Analysis of error data revealed no significant effects, all *F*(1,15) ≤ 1.63, *p* ≥ .221.


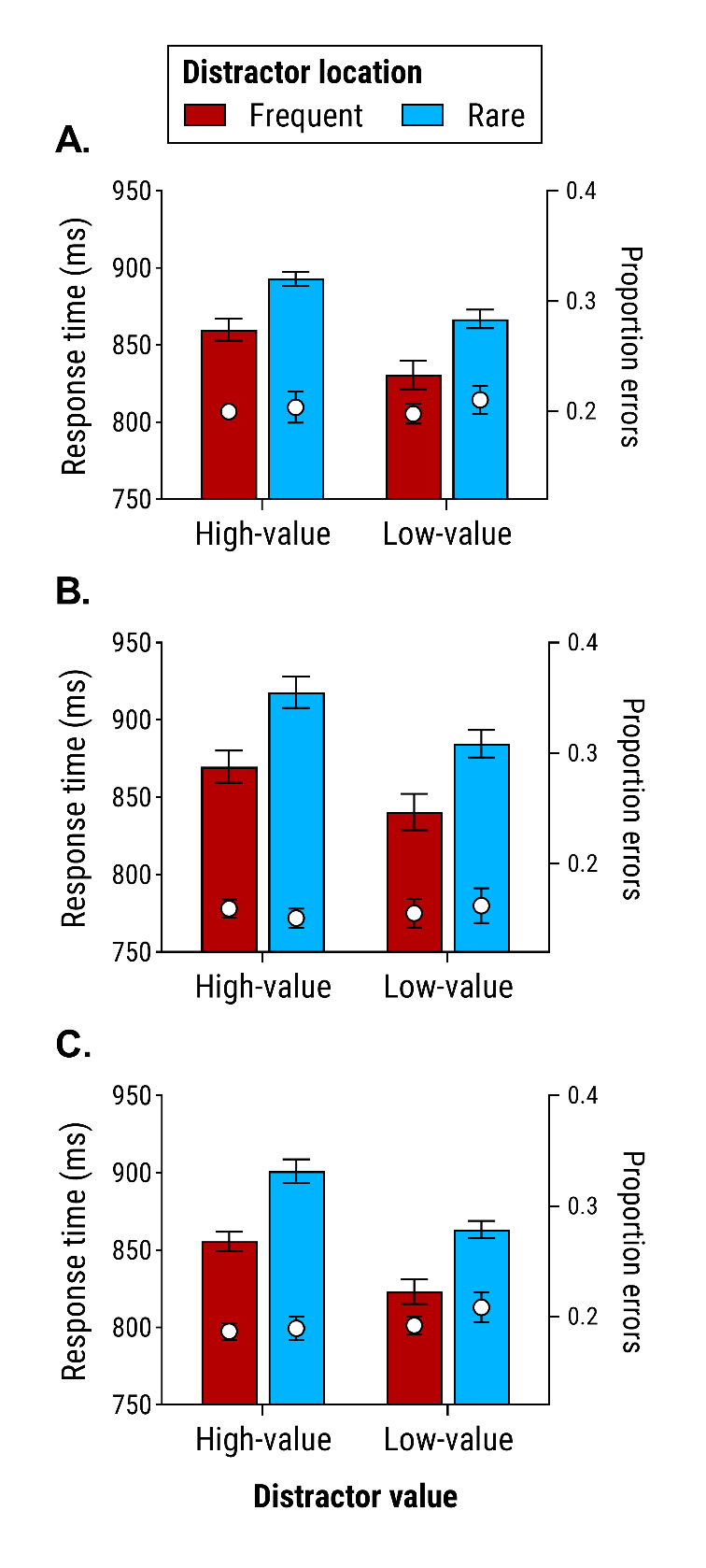


**Figure S1A.** Mean response times and proportion of errors for trials with a colour-singleton distractor in Experiment 1, as a function of the value of the reward signalled by the distractor, and the location of that distractor. Data are shown for participants who failed to answer each of the three ‘explicit awareness’ questions correctly: the top/middle/bottom question **(A)**; the left/central/right question **(B)**; and the specific location question **(C)**. Bars show mean response time, superimposed white circles show mean proportion of errors, and error bars show within-subjects standard error of the mean.

# Experiment 2

In the test of explicit awareness following the search task of Experiment 2, 13 participants of the 41 participants correctly selected the frequent locations of the high- and low-value distractors. Figure S2 shows RT and error data for the remaining 28 participants who chose the incorrect location for one or both distractors. Data are grouped according to whether the distractor appeared in its own most frequent location (i.e., high-value distractor in frequentHigh location; low-value distractor in frequentLow location: labelled the *match* condition), or in the most frequent location of the other type of distractor (high-value distractor in frequentLow location and vice versa: *mismatch* condition), or in one of the rare locations. As in the main text, for this analysis we excluded trials in which the target appeared at either the frequentHigh or frequentLow location, i.e., for all trials in this analysis, the target appeared at a ‘rare’ location.

RT data were analysed via 2×3 ANOVA with factors of distractor value (high-value vs. low-value) and location (match, mismatch, rare). This revealed a main effect of distractor value, *F*(1,27) = 15.7, *p* < .001, η*_p_*^2^ = .368, with slower responses for high-value than low-value trials, and a main effect of location, *F*(2,54) = 4.65, *p* = .014, η*_p_*^2^ = .147. There was no significant value × location interaction, *F*(2,54) = 0.20, *p* = .817, η*_p_*^2^ = .007. Analysis of error data revealed no significant effects, all *F* < 1.


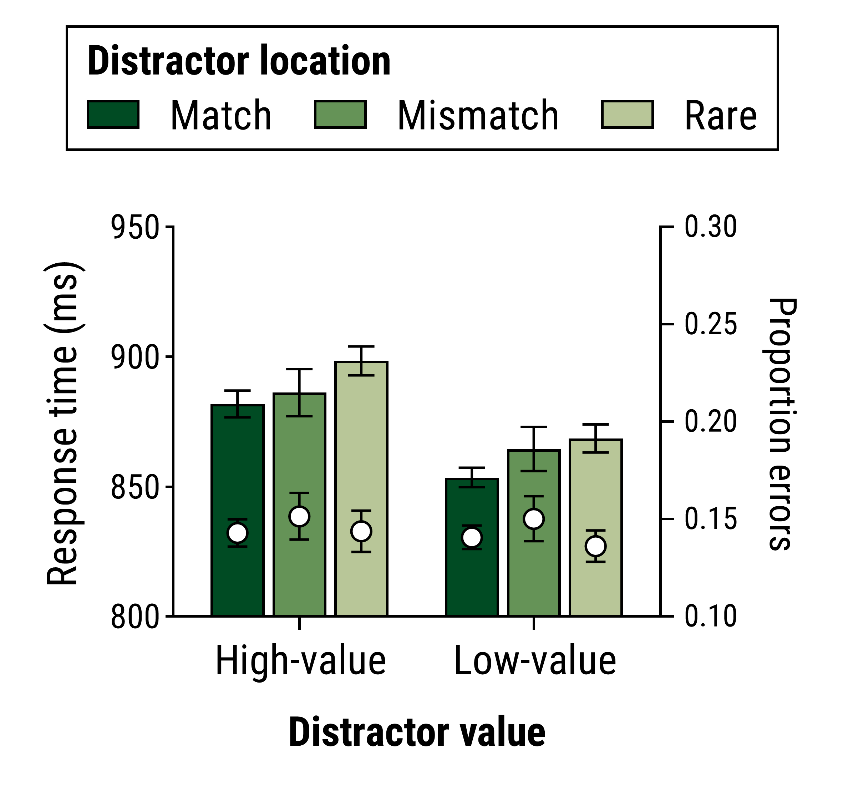


**Figure 4.** Mean response times and proportion of errors for trials with a colour-singleton distractor in Experiment 2, as a function of the reward value of the distractor, and its location: whether the distractor appeared in its own most frequent location (i.e., high-value distractor in frequentHigh location; low-value distractor in frequentLow location: labelled *match*), or in the most frequent location of the other type of distractor (high-value distractor in frequentLow location and vice versa: *mismatch*), or in one of the rare locations. For comparison, the figure also shows mean performance on distractor-absent trials.
